# Application of three-dimensional imaging software to map carcinomatosis in recurrent ovarian cancer

**DOI:** 10.1093/jscr/rjae188

**Published:** 2024-04-01

**Authors:** Ana Gomes da Costa, Diogo Albergaria, Joana Almeida, Mónica Nave, Joana Oliveira, Ana Catarino, Dennis S Chi, João Casanova

**Affiliations:** Gynecologic Oncology Unit, Hospital da Luz, Lisboa, Avenida Lusíada, 100,1500-065, Portugal; Department of General Surgery, Hospital da Luz, Lisboa, Avenida Lusíada, 100,1500-065, Portugal; Gynecologic Oncology Unit, Hospital da Luz, Lisboa, Avenida Lusíada, 100,1500-065, Portugal; Department of Radiology, Hospital da Luz, Lisboa, Avenida Lusíada, 100,1500-065, Portugal; Gynecologic Oncology Unit, Hospital da Luz, Lisboa, Avenida Lusíada, 100,1500-065, Portugal; Department of Oncology, Hospital da Luz, Lisboa, Avenida Lusíada, 100,1500-065, Portugal; Department of Anesthesiology, Hospital da Luz, Lisboa, Avenida Lusíada, 100,1500-065, Portugal; Gynecologic Oncology Unit, Hospital da Luz, Lisboa, Avenida Lusíada, 100,1500-065, Portugal; Department of Pathology, Hospital da Luz, Lisboa, Avenida Lusíada, 100,1500-065, Portugal; Gynecology Service, Department of Surgery; Memorial Sloan-Kettering Cancer Center, New York, 1275 York Ave, New York, NY 10065, USA; Gynecologic Oncology Unit, Hospital da Luz, Lisboa, Avenida Lusíada, 100,1500-065, Portugal

**Keywords:** 3D modeling, image processing, carcinomatosis, secondary cytoreductive surgery

## Abstract

The treatment of recurrent ovarian cancer has been based on systemic therapy. The role of secondary cytoreductive surgery has been addressed recently in several trials. Imaging plays a key role in helping the surgical team to decide which patients will have resectable disease and benefit from surgery. The role of staging laparoscopy and several imaging and clinical scores has been extensively debated in the field. In other surgical fields there have been reports of using 3D imaging software and 3D printed models to help surgeons better plan the surgical approach. To the best of our knowledge, we report the first case of a patient with recurrent ovarian cancer undergoing 3D modeling before secondary cytoreductive surgery. The 3D modeling was of most value to evaluate the extension of the disease in our patient who underwent a successful secondary cytoreductive surgery and is currently free of the disease.

## Introduction

Ovarian cancer remains one of the most challenging diseases in gynecologic oncology and ~80% of patients with advanced disease will relapse [1–4]. In recurrent ovarian cancer (ROC) the success of cytoreductive surgery is mostly dependent on careful selection of the patients who are eligible for surgery. The survival benefit may depend on selection criteria that include algorithms such as the Arbeitsgemeinschaft Gynakologische Onkologie score, the criteria defined by Chi *et al*., and the International Model score [2–5].

Imaging has an important role in the evaluation of ROC. Computed tomography (CT) scans, magnetic resonance imaging (MRI), and positron emission tomography can be used and ultimately a diagnostic laparoscopy can be performed [1, 5, 6].

The use of three-dimensional (3D) imaging models has been reported for hepatic and colorectal cancers. The 3D models can be generated from CT scans and MRI scans. [6–11].

The 3D models elaborated by Cella Medical solutions® enabled us to obtain 360° views of the tumor and its relation or infiltration to adjacent organs such as the abdominal and pelvic organs. It is also possible to evaluate the relation between the tumor and the vascular structures and to calculate the disease volume and the peritoneal cancer index (PCI).

We report a case of an ovarian cancer relapse in which 3D modeling was a valuable resource to evaluate the extent of the disease and for surgical planning.

## Case report

**Figure 1:**
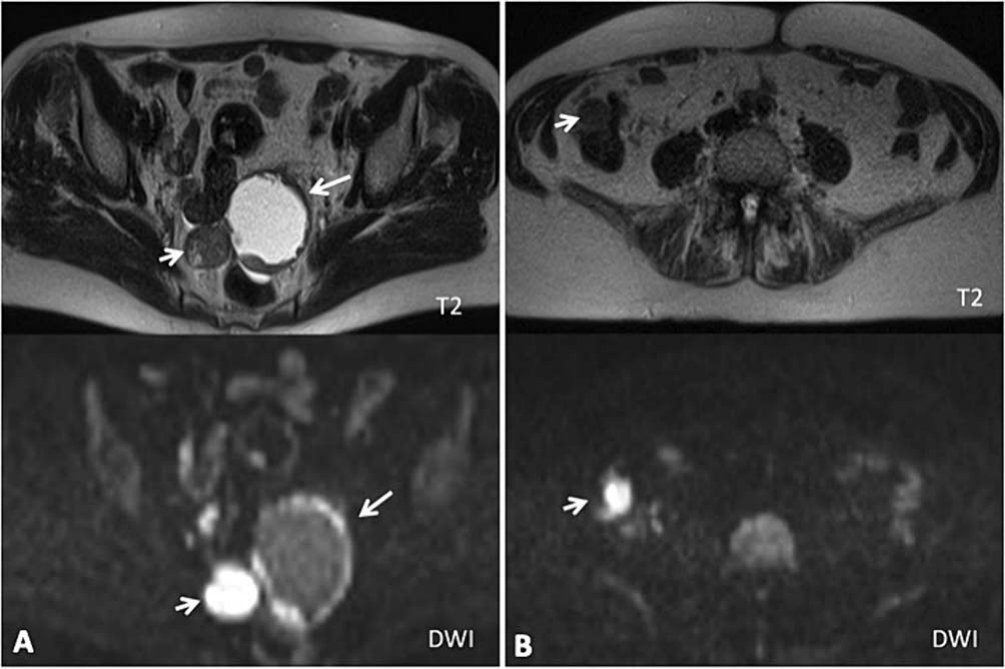
(A) Axial MRI imaging showing the pelvic tumor implants; (B) axial MRI imaging showing the implants in the right colon (diffusion weighted imaging).

**Figure 2:**
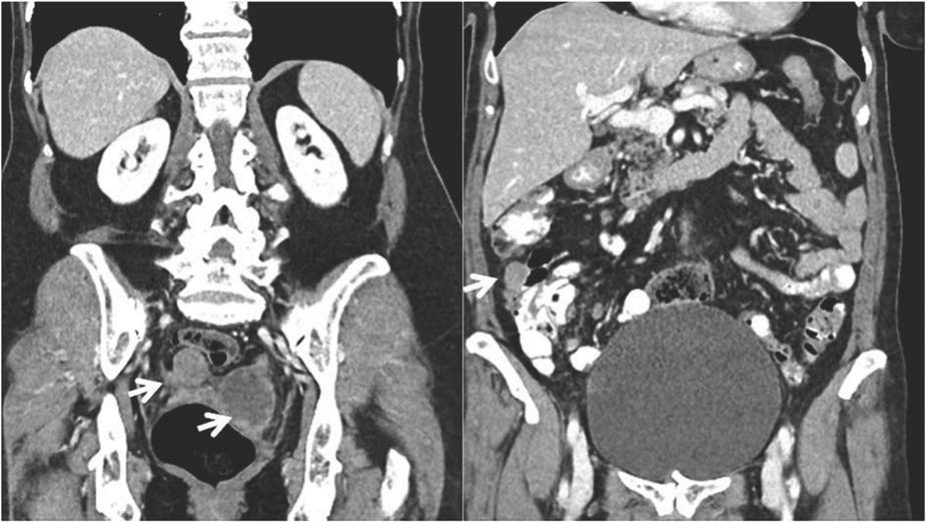
Coronal CT imaging showing the pelvic tumor implants and in the right colon.

A 68-year-old patient, initially diagnosed with a FIGO stage IIA fallopian tube carcinoma, was under active follow-up in the Gynecologic Oncology Clinic at our institution since 2020. She underwent primary cytoreductive surgery in August 2019 (hysterectomy, bilateral salpingo-oophorectomy, omentectomy, pelvic and para-aortic lymphadenectomy, without any residual macroscopic disease) and completed six cycles of postoperative chemotherapy (carboplatin and paclitaxel) in January 2020. She was free of disease until May 2022 (disease free interval—26 months) when her CA 125 began to rise (52 U/L). CT and MRI scans showed splenic and subcapsular hepatic implants, two metastatic pelvic nodules with 31 and 58 mm next to the sigmoid colon and rectum, and several mesenteric implants in the descendent colon (Figs 1 and 2).

The case was discussed in a multidisciplinary team meeting and a laparoscopy was performed to evaluate the extent of the disease. Laparoscopy confirmed a pelvic mass adherent to the vagina and rectum, some mesenteric implants that were resectable but the right colon and upper abdomen (including the diaphragm) were not possible to be adequately evaluated due to severe adhesions.

**Figure 3:**
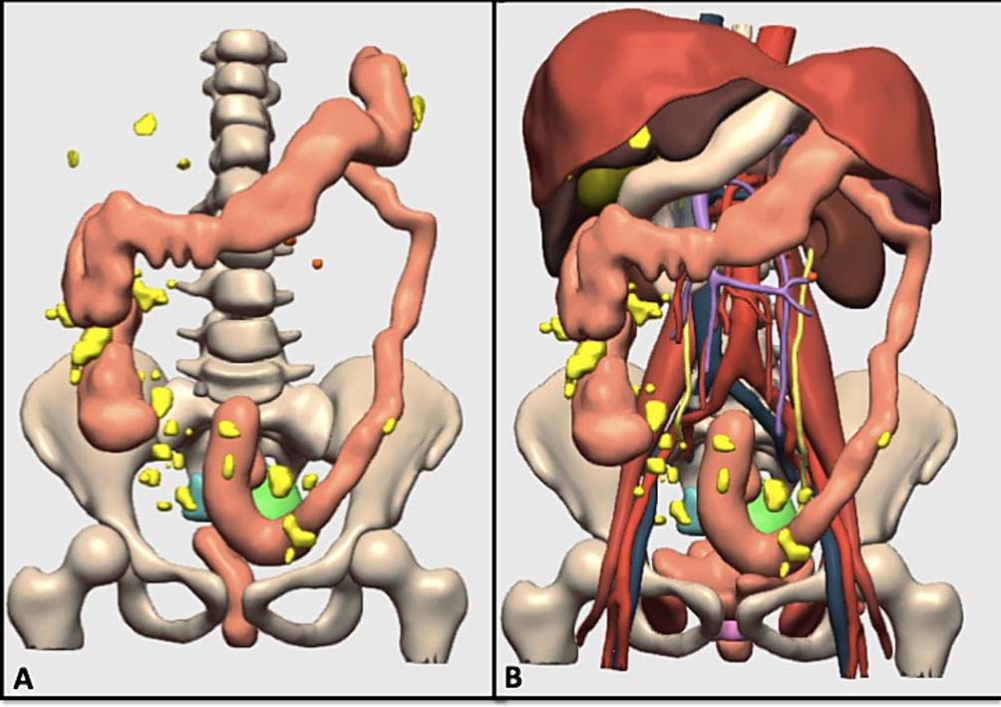
(A) 3D model of the tumor and carcinomatosis; the generated model includes the carcinomatosis (yellow), possible adenopathies (orange), cystic pelvic lesion (green), solid pelvic lesion (blue), rectum and colon (brown); (B) 3D model of the tumor, carcinomatosis, and anatomical structures; the generated model includes the carcinomatosis (yellow), arterial vessels (red) venous vessels (blue), portal vessels (purple), liver (dark brown), stomach and duodenum (white), biliary bladder (green), spleen (dark purple), pancreas (gray).

**Figure 4:**
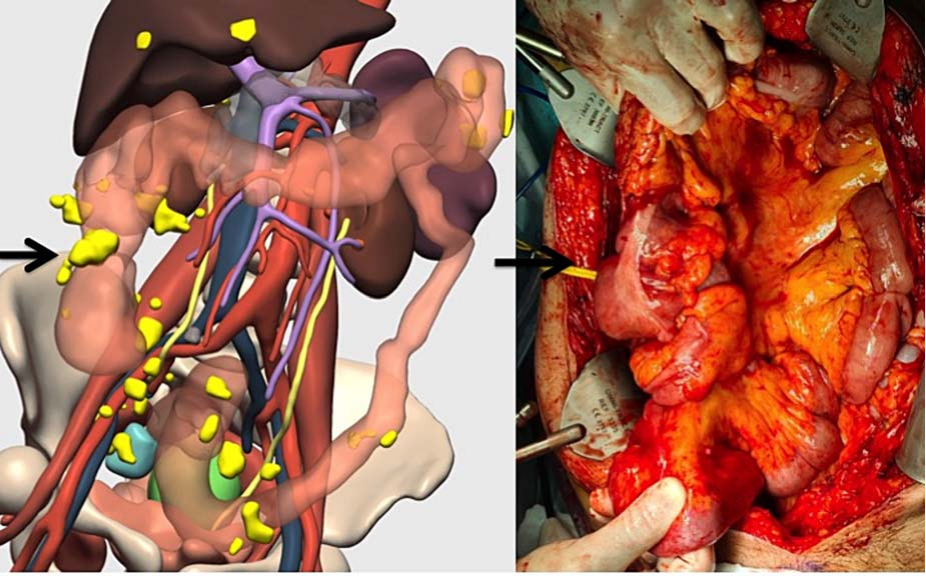
Correlation between the 3D model and surgical findings on the right colon and tumor implants (arrows).

Using the MRI scans, a 3D reconstruction of the findings was performed by a multidisciplinary team consisting of clinical and technical specialists from Cella Medical Solutions using 3D digital image reconstruction software (‘3D-MSP’, Cella Medical Solutions©, Murcia, Spain). Advanced and complex techniques of image processing are involved while creating 3D models. First, fully automatic algorithms are used to enhance image quality and segment anatomical structures of interest. Then 2D masks are generated and used to reconstruct a 3D mesh of each individual anatomical structure (Figs 3 and 4).

The evaluation of these 3D models confirmed the tumor implants on the splenic surface, the hepatic capsule, the sigmoid colon/rectum, and on the vagina. The 3D models also showed a 2 cm-right diaphragmatic implant and right colon implants and determined a PCI value of 15.

The patient had a performance status of 0 and was considered eligible for a secondary cytoreductive surgery. The surgery included the following procedures: resection of implants on the diaphragm, hepatic capsule, Morrison’s Pouch, small bowel mesentery, and large bowel. Visceral resections included a splenectomy, a right hemicolectomy with ileal resection (80 cm length), and a rectosigmoid resection (Fig. 4). Suspicious pelvic lymph nodes were also resected. A protective ileostomy was performed. Complete gross resection was achieved.

After surgery, the patient was admitted for 3 days in the intensive care unit and the immediate postoperative period was uneventful. However, on the 12th postoperative day the patient underwent exploratory laparotomy due to persistent fever, pneumoperitoneum, and elevation of inflammatory parameters. A right diaphragmatic defect was found and repaired and a right thoracic drain was placed. There were no other complications. The patient was discharged on the 20th postoperative day and resumed chemotherapy (Carboplatin/Paclitaxel). Currently there is no clinical or imaging evidence of a relapse.

## Discussion

There is no consensus regarding the optimal treatment for ROC as the dilemma between secondary cytoreduction and systemic therapy remains.

Our case shows that the development of 3D models may be helpful to evaluate patients with ROC for secondary surgery. In this clinical case a CT, an MRI, and a diagnostic laparoscopy were performed to evaluate the extent of the disease. Unfortunately, it was not possible to assess with certainty the right colon and the right upper abdomen. The 3D modeling was of most value to evaluate the extension of the disease that was not adequately evaluated by laparoscopy. With adequate surgical planning, both the surgeon and the patient were fully prepared for the extent and complexity of the operative procedures.

The use of 3D models has many advantages, which include the evaluation of anatomical variants, safety margins, and a better understanding of the relation between the tumor implants and the abdominal and pelvic organs. In cytoreductive surgery for ovarian cancer it can be helpful prior to surgery to evaluate the extension of the disease and to plan organ resections such as the bowel. Therefore, the study of the 3D models can be helpful to reduce the intraoperative complications and to prepare the patients for the extent of the surgery [5–12].

There are already multiple studies demonstrating the value of virtual reconstruction and 3D printing models for surgical planning in multiple areas, including oncology. However, when considering the use of this technique we should also bear in mind its cost and the potential advantages for each patient. Therefore, more studies are needed in this area to rely on these models for cancer staging, which could reduce the need for diagnostic laparoscopies and surgical complications, especially in complex surgical scenarios.

## Author contributions

Ana Gomes da Costa (Conceptualization, Writing—Original Drafting, Review and Editing), Diogo Albergaria (Writing—Review and Editing), Joana Almeida (Conceptualization, Writing—Review and Editing), Mónica Nave (Writing—Review and Editing), Joana Oliveira (Writing—Review and Editing), Ana Catarino (Conceptualization, Writing—Review and Editing), Dennis S. Chi (Writing—Review and Editing), João Casanova (Conceptualization, Supervision, Writing—Review and Editing).

## Conflict of interest statement

The authors declare no conflict of interests.

## Funding

None declared.

## Patient consent

Written informed consent was obtained from the patient for publication of this case report and accompanying images.
